# The burden and treatment of non-communicable diseases among healthcare workers in sub-Saharan Africa: a multi-country cross-sectional study

**DOI:** 10.3389/fpubh.2024.1375221

**Published:** 2024-05-13

**Authors:** Sophie Alice Müller, Kelly Elimian, Jean Florent Rafamatanantsoa, Felix Reichert, Francis Mosala, Lena Böff, Sounan Fidèle Touré, Idesbald Boone, Lantonirina Ravaoarisoa, Sagesse Nduenga, Giuseppina Ortu, Francisco Pozo-Martin, Sara Tomczyk, Tim Eckmanns, Tochi Okwor, Chantal Akoua-Koffi, Bamourou Diané, Zely Arivelo Randriamanantany, Steve Ahuka, Chinwe Lucia Ochu, Charbel El-Bcheraoui

**Affiliations:** ^1^Robert Koch Institute, Berlin, Germany; ^2^Nigeria Centre for Disease Control and Prevention, Abuja, Nigeria; ^3^Department of Global Public Health, Karolinska Institutet, Stockholm, Sweden; ^4^Laboratoire d’Analyses Médicales Malagasy, Antananarivo, Madagascar; ^5^Institut National de la Recherche Biomédicale, Kinshasa, Democratic Republic of Congo; ^6^Center Hospitalier et Universitaire de Bouaké, Bouaké, Côte d'Ivoire; ^7^Global Health Consultant, London, United Kingdom

**Keywords:** non-communicable diseases, sub-Sahara Africa, hypertension, diabetes, healthcare workers

## Abstract

**Introduction:**

Non-communicable diseases (NCDs), the leading cause of death globally, are estimated to overtake communicable diseases in sub-Sahara Africa, where healthcare workers (HCWs) play a crucial role in prevention and treatment, but are in extreme shortage, thereby increasing the burden of NCDs among this specific population. To provide evidence for policy-making, we assessed the NCD burden, associated factors and treatment among HCWs in four sub-Saharan African countries.

**Materials and methods:**

We conducted a cross-sectional study across four sub-Saharan African countries [Côte d'Ivoire (CIV), Democratic Republic of the Congo (DRC), Madagascar (MDG), and Nigeria (NIG)] between February and December 2022. In a standardized questionnaire, sociodemographic, chronic disease and treatment data were self-reported. We estimated the prevalence of (1) at least one chronic disease, (2) hypertension, and used backward elimination logistic regression model to identify risk factors.

**Results:**

We recruited a total of 6,848 HCWs. The prevalence of at least one chronic disease ranged between 9.7% in NIG and 20.6% in MDG, the prevalence of hypertension between 5.4% in CIV and 11.3% in MDG. At most, reported treatment rates reached 36.5%. The odds of each of both outcomes increased with age (at least one chronic disease adjusted odds ratio: CIV: 1.04; DRC: 1.09; MDG: 1.06; NIG: 1.10; hypertension: CIV: 1.10; DRC: 1.31; MDG: 1.11; NIG: 1.11) and with BMI (at least one chronic disease: CIV: 1.10; DRC: 1.07; MDG: 1.06; NIG: 1.08; hypertension: CIV: 1.10; DRC: 1.66; MDG: 1.13; NIG: 1.07). Odds of both outcomes were lower among males, except in CIV. In NIG, the odds of both outcomes were higher among medical doctors and odds of hypertension were higher among those working in secondary care. In MDG, working in secondary care increased and working as auxiliary staff decreased the odds of at least one chronic disease.

**Conclusion:**

The prevalence of self-reported chronic disease varied across the four sub-Saharan countries with potentially very low treatment rates. We identified several individual (age, sex, and BMI) and occupational (profession, level of healthcare) factors that influence the odds of NCDs. These factors should be taken into account when developing interventions addressing the burden and management of NCDs among HCWs.

## Introduction

Non-communicable diseases (NCDs) are the leading cause of deaths, resulting in 41 million deaths annually and accounting for more than three-quarters of all deaths globally (1). NCDs disproportionately affect people in low- and middle-income countries, where more than 75% of global NCD deaths and 86% of premature deaths (death between 30 and 70 years) occur (1, 2). In sub-Saharan Africa, NCDs have been projected to overtake communicable, maternal, neonatal, and nutritional diseases combined as the leading cause of mortality by 2030 (3).

To tackle the burden of NCDs, the sustainable development goal (SDG) target 3.4 is set to reduce premature mortality from NCDs by one-third through prevention and treatment (4). To achieve this reduction, it is essential that patients have a good understanding of their disease and have appropriate access to effective treatment. Healthcare workers (HCWs), by educating and caring for patients, play a crucial role in the prevention and treatment of NCDs. Despite this crucial role, working as a HCW is recognized as a high-risk occupation, as HCWs face regularly physical and mental stress from work shifts, overtime, as well as pressure through provision of care under life-and-death circumstances (5). Working night shifts for example leads to a lack of time to prepare healthy meals and is linked to dyslipidemia (6). When it comes to risk behaviors, a study from Nigeria showed low physical activity levels among HCWs (7). Furthermore, HCWs in general continue to consume tobacco at high rates (8). These risk factors can be exacerbated by mental health issues. Indeed, HCWs are at higher risk for suicide (9) and mental stress *per se* is linked to an increased risk for mortality due to associated physical health issues (10). A systematic review synthesizing evidence on HCWs in Africa found that 49% of frontline HCWs suffered from a mental health symptom during the COVID-19 pandemic (11).

HCWs are in high demand globally, but the most severe shortage is experienced in the African region. With an average of 1.3 HCWs per 1,000 population, the African region is far below the average of 4.5 per 1,000 required for the SDGs (12) with a forecasted shortage of 6.1 million by 2030 (13). This unprecedented shortage is further exacerbated when HCWs are absent for long periods due to chronic diseases and results in excessive workload, burnout, and mental stress for the available few. Data on the health status of HCWs across the African region are scare, but indicate the need for detailed epidemiological data to address the burden of NCDs. Across sub-Saharan Africa many NCDs are rising (14), but data are hardly available on country level (3). Recent studies from Ghana, Mozambique, and Zimbabwe reported a double burden of NCDs and infectious diseases in the general population (15–17). A scoping review from 2019 found hypertension as the most commonly reported NCD followed by obesity and diabetes (18) with a prevalence range from 17.5 to 37.5% among HCWs (19). Small studies suggest that 16% of HCWs in Ghana and 37.1% in Nigeria suffer from hypertension (20, 21), whereas in South Africa, 73% of HCWs have been found to be overweight or obese (22). A study from Nigeria found, that only about 40% of hypertensive HCWs were on therapy (21), underlining the need for appropriate management of NCDs in HCWs.

In the context of constrained resources and shortage of HCWs, information about the prevalence and management of NCDs in HCWs is essential to treat and protect HCWs, to promote them as educators and role models of healthy behaviors for patients, as well as to safeguard them as an essential workforce that provides healthcare for patients, ultimately contributing to the achievement of the SDG target 3.4.

Within the framework of a larger study on the prevalence of SARS-CoV-2 and vaccine coverage in HCWs, we assessed the self-reported burden of NCDs, associated factors and their treatment among HCWs in four African countries in an attempt to provide health authorities and decision makers with the evidence to help protect HCWs.

## Materials and methods

### Study design and setting

Data on the prevalence of self-reported NCDs among HCWs were collected through a transnational cross-sectional multisite survey. As part of a larger study on the burden of COVID-19, data collection took place between February and December 2022 as a collaboration between the Robert Koch Institute, Berlin Germany, and four institutions in sub-Saharan partner countries: Center Hospitalier et Universitaire de Bouaké (Côte d'Ivoire), Institut National de la Recherche Biomédicale (Democratic Republic of the Congo), Laboratoire d’Analyze Médicale Malagasy (Madagascar), and Nigeria Center for Disease Control and Prevention (Nigeria).

The health systems in each country can be described as follows:

In Côte d'Ivoire, there are primary, secondary, and tertiary level facilities in the public health sector. The secondary level of care consists of district and regional hospitals. The tertiary level of care provides more specialized care and includes university hospitals. At all three levels, there are also private facilities (23).
In the Democratic Republic of the Congo, the health system of each region is structured into health zones and smaller health areas. Within each zone, there is a public healthcare facility serving as reference hospital for the zone. This could be a primary care or secondary care level (one per region). In all health areas, there is at least one public health center at the primary care level and often several private healthcare facilities.
In Madagascar, there are three tiers in the public health sector: (1) community-based primary healthcare facilities (HCFs), (2) district referral hospitals, and (3) regional referral hospitals and university hospitals. In addition, at primary and secondary level of care, there are private facilities (24).
In Nigeria, the health system has three levels of care (primary, secondary, and tertiary). At each level of care, there are both public health sector facilities and private (including faith-based organizations) facilities (25).

### Sampling

In the context of a larger study, the sample size for the cross-sectional survey was calculated based on the main outcome of seroprevalence of SARS-CoV-2, estimating a seroprevalence of 50%, with a confidence level of 95% and multiplied by a design effect of 2 for multistage sampling equaling 761 HCWs.

The study population included all formally employed and informally engaged (e.g., medical and nursing students) HCWs as well as allied and auxiliary health workers, e.g., administration staff and security personnel. This inclusive approach was based on the WHO guideline (26). In this guideline, “health management and support personnel” consist of health service managers, such as administrative staff, and of services workers, such as security guards. This inclusive approach reflects the unique work environment within HCFs and gives us the possibility to assess occupational risk factors across all kinds of healthcare settings. Inclusion criteria can be summarized as the following: HCW meets the above definition; is over 18 years old and is willing and able to participate.

In Côte d'Ivoire, the study was carried out in the regions of Gbêkê, Hambol, and Cavally-Guémon. Due to logistic constraints, a total of 13 public healthcare facilities (HCFs) were purposively selected based on an ongoing collaboration with the Center Hospitalier et Universitaire of Bouaké. For every region, the selected HCFs in Gbêke represent 15% of the total workforce, in Hambol 37% and in Cavally-Guémon 45%. Within each HCF, all available HCWs were invited to participate.

In Madagascar, the Democratic Republic of the Congo, and Nigeria, we aimed for a multistage random sampling with subregions as the primary sampling unit, HCF as the secondary sampling unit and HCWs as the tertiary sampling units. However, areas that were determined unsafe (e.g., potential conflict) or inaccessible (e.g., no available road) were excluded from selection.

In the Democratic Republic of the Congo, the study was conducted in the region of Kwilu and Kwango because of scarcity of seroprevalence data in these two regions. Within each region, administrative health zones were stratified by availability of secondary care. Within selected zones, public HCFs in up to ten health areas within a radius of 20 km from the site of the main reference hospital were randomly selected. HCWs were stratified by direct/indirect patient contact and randomly selected.

In Madagascar, four regions, and within these regions, eleven districts were purposively selected. In each district, region capitals were selected as a priority, followed by district capitals, to ensure that all levels of the HCFs were represented (including those that were regional reference hospitals or university hospitals present in region capitals). In each capital, at least one HCF at the highest level of the healthcare structure was sampled whereas at the primary healthcare level, several HCFs were randomly selected from a list of healthcare structures. All personnel in each HCF were invited to participate.

In Nigeria, two states, River State and the Federal Capital Territory (FCT), were purposively selected based on the COVID-19 burden and logistical considerations such as established partnerships with local partners. In each state, local government areas (LGAs) were stratified according to those with or without a tertiary care facility and then randomly selected. HCFs in the selected LGAs were further stratified by level of care and ownership (public or private) and then randomly selected. Lastly, HCWs were selected by a simple random sampling approach.

### Data collection and analysis

The questionnaire was developed with input from all participating country partners. The following steps were undertaken (1) item generation: a literature review as well as expert consultation was performed to identify potential items, (2) scale purification: the initial questionnaire was administered to a peer research group to assess clarity or refine wording, (3) validity: in collaboration with a survey design expert team and the local research team the content validity was evaluated. In accordance with Boynton et al. (27), the questionnaire was standardized across countries for the entire multisite study, meaning that every participant was asked the same question in a uniform manner. In each of the four countries, data collectors participated in a 3-day workshop to familiarize themselves with the survey questionnaire, methodology, and the software used for data collection. Consecutively, the survey was piloted for recruitment, functionality of the software, and applicability of the questionnaire in a sample of HCFs over 2 days. By following this process, we made sure that the overall standardized questionnaire could be applied across all settings.

Local interviewers were trained on basic interviewing techniques and the use of the questionnaire. The questionnaire included sociodemographic characteristics, tobacco consumption, working environment, and self-reported chronic disease and treatment.

Our main outcome variables were: (1) one or more self-reported chronic disease and (2) self-reported hypertension. We defined NCDs following the *Lancet non-communicable diseases and injuries Commission* (28), including conditions such as diabetes, heart disease, chronic lung disease, or cancer, but excluded sickle cell disorder as an inherited disease. If a condition reported by study participants was not named by the commission, we only included it if risk factors assessed in our study could influence this condition. We used self-reported weight and height to calculate body mass index (BMI) as weight (kg)/height (m^2^). Participants were classified into three groups: (1) underweight or normal weight: BMI < 25.0; (2) overweight: BMI within 25.0–29.9; or (3) obese: BMI greater than or equal to 30.0. Covariates were selected from the initial questionnaire based on medical relevance and scientific evidence (28). Regarding treatment, we asked participants if they took angiotensin-converting enzyme inhibitors, corticosteroids, non-steroidal anti-inflammatory, or anti-rheumatic drugs.

Trained interviewers administered the standardized questionnaire to each individual HCW using Open Data Kit (ODK) forms and directly entered responses on study designated tablets. Questionnaire data were stored on ODK servers (29) and data checks, cleaning and analysis were conducted including packages “dplyr” “tidyverse” (30) with the statistical software R version 4.1.2 (2021-11-01) (31), and STATA in version 17 (StataCorp. 2021. *Stata Statistical Software: Release 17*. College Station, TX: StataCorp LLC).

Characteristics of study participants were descriptively analyzed. Prevalence estimates for each outcome were calculated by country. To identify potential risk factors associated with each outcome, logistic regression was used including a backward elimination procedure to identify regressors retained in each model. For each outcome variable, separate models were run by country. Initially included variables (sex, age, level of care, BMI, smoking, and profession) were consecutively excluded based on statistical significance, and on the Bayesian-Information-Criterion. The final model was checked for miss-specification, goodness of fit (32), multicollinearity as well as influential observations and adapted accordingly. Odds ratio, as well as adjusted Odds Ratios (AOR) and 95% confidence intervals (95% CI) were reported for each country specific model. As the sampling strategy differed by country, and a stratified random sampling design could not be systematically implemented, we applied direct statistical modeling without further stratification.

### Ethical considerations

Ethics approval was received from the ethics committee of Berlin Charité (Number EA2/248/21) and from national ethics committees of each partner country (Côte d'Ivoire: 216-21/MSHPCMU/CNESVS-km, Democratic Republic of the Congo: ESP/CE/22B/2022, Madagascar: 12 MSANP/SG/AMM/CERBM, Nigeria: NHREC/01/01/2007-29/11/2021). Written informed consent was obtained from each participant prior to data collection. Only limited trained study staff had access to the data on tablets and data were (pseudo-)anonymized upon analysis.

## Results

### Characteristics of study participants

In total, 27,357 HCWs worked in the selected districts (Côte d'Ivoire: 3,820; Democratic Republic of the Congo: 2,540; Madagascar 5,141; Nigeria 15,846), out of which we recruited a total of 6,848 (25.0%) HCWs across the four countries (Table 1). Frequent reasons for non-response were lack of time and discomfort of sample collection The majority of the HCWs were females (60.8%, 4,162/6.848) with a median age of 37 years. Obesity combined with overweight was most prevalent in Nigeria (60.7%, 1,390/2,291), and least prevalent in Madagascar (30.2%, 848/2,804). Only 10.7% of HCWs (730/6.848) reported to currently or previously smoke.

**Table 1:** Characteristics of the study participants by country.

Characteristic	All countries	Côte d'Ivoire	DRC	Madagascar	Nigeria
	(*n* = 6,848)	(*n* = 518)	(*n* = 1,232)	(*n* = 2,804)	(*n* = 2,291)
	Frequency (%)	Frequency (%)	Frequency (%)	Frequency (%)	Frequency (%)
Sex
Female	4,162 (60.8)	302 (58.3)	633 (51.4)	1,722 (61.4)	1,505 (65.7)
Male	2,681 (39.2)	216 (41.7)	599 (48.6)	1,082 (38.6)	784 (34.2)
Missing	5 (0.1)	0	0	0	5 (0.2)
Age
Median (IQR)	37 (29–45)	34 (28–42)	40 (32–50)	34 (27–45)	38 (31–45)
Missing	5 (0.2)	0	0	0	5 (0.2)
Education
Primary	222 (3.2)	25 (4.8)	21 (1.7)	124 (4.4)	52 (2.3)
Secondary	1,605 (23.4)	206 (39.8)	290 (23.5)	762 (27.2)	347 (15.1)
Higher	4,895 (71.5)	281 (54.2)	841 (68.3)	1,908 (68.0)	1,865 (81.4)
Missing	126 (1.8)	6 (1.2)	80 (6.5)	10 (0.4)	30 (1.3)
BMI
Under/normal weight (<25)	3,781 (55.2)	246 (47.5)	812 (65.9)	1,956 (69.8)	767 (33.5)
Overweight (25–29.9)	1,937 (28.3)	190 (36.7)	276 (22.4)	693 (24.7)	778 (34.0)
Obesity (≥ 30.0)	944 (13.8)	82 (15.8)	95 (7.7)	155 (5.5)	612 (26.7)
Missing	186 (2.7)	0	49 (4.0)	0	137 (6.0)
Smoking
Never smoked	6,078 (88.8)	473 (91.3)	1,045 (84.8)	2,399 (85.6)	2,161 (94.3)
Current/past smoker	730 (10.7)	41 (7.9)	178 (14.4)	397 (14.2)	114 (5.0)
Missing	40 (0.6)	4 (0.8)	9 (0.7)	8 (0.3)	19 (0.8)
Profession
Administrative staff	1,014 (14.8)	85 (16.4)	84 (6.8)	403 (14.4)	442 (19.3)
Medical doctor	515 (7.5)	30 (5.8)	70 (5.7)	244 (8.7)	171 (7.5)
Paramedics with higher education	3,119 (45.5)	172 (33.2)	745 (60.5)	1,533 (54.7)	669 (29.2)
Paramedics with lower education	896 (13.1)	148 (28.6)	175 (14.2)	85 (3.0)	488 (21.3)
Auxiliary staff	1,149 (16.8)	73 (14.1)	158 (12.8)	520 (18.5)	398 (17.4)
Missing	155 (2.3)	10 (1.9)	0	19 (0.7)	126 (5.5)
Level and type of care
Primary hospital	584 (8.5)	NA	385 (31.3)	199 (7.1)	NA
Primary non-hospital	1,810 (26.4)	326 (62.9)	653 (53.0)	403 (14.4)	392 (17.1)
Private	1,350 (19.7)	NA	NA	850 (30.3)	500 (21.8)
Secondary	1,454 (21.2)	192 (37.1)	194 (15.7)	530 (18.9)	538 (23.5)
Tertiary	1,611(23,5)	NA	NA	820 (29.2)	791 (34.5)
Missing	75 (1.1)	0	0	2 (0.1)	73 (3.2)
Chronic condition
Hypertension	603 (8.8)	28 (5.4)	97 (7.9)	317 (11.3)	161 (7.0)
Diabetes	111 (1.6)	9 (1.7)	26 (2.1)	51 (1.8)	25 (1.1)
Asthma	128 (1.9)	20 (3.9)	16 (1.3)	63 (2.2)	29 (1.3)
Heart disease	117 (1.7)	5 (1.0)	12 (1.0)	92 (3.3)	8 (0.4)
Diabetes	111 (1.6)	9 (1.7)	26 (2.1)	51 (1.8)	25 (1.1)
Hepatological disease	59 (0.9)	4 (0.8)	1 (0.1)	51 (1.8)	3 (0.1)
Lung disease	24 (0.4)	4 (0.8)	5 (0.4)	11 (0.4)	4 (0.2)
Neurological disease	22 (0.3)	1 (0.2)	4 (0.3)	8 (0.3)	9 (0.4)
Kidney disease	15 (0.2)	0 (0)	2 (0.2)	12 (0.4)	1 (0.0)
Hematological disease	10 (0.1)	4 (0.8)	0 (0)	5 (0.2)	1 (0.0)
Cancer	8 (0.1)	0 (0)	2 (0.2)	3 (0.1)	3 (0.1)
Other NCD	140 (2.0)	8 (1.5)	10 (0.8)	113 (4.0)	9 (0.4)

DRC, Democratic Republic of Congo; BMI, Body mass index; IQR, Interquartile range; NA, Not applicable; NR, Not reported.

### Prevalence of at least one chronic disease and associated risk factors

The proportions of HCWs reporting at least one chronic (Table 1) disease by country were 9.7% (222/2291; 27/2291 reporting multiple diseases) in Nigeria, 11.8% (145/1,232; 26/1,232 reporting multiple diseases) in the Democratic Republic of the Congo, 13.5% (70/518, 11/518 reporting multiple diseases) in Côte d'Ivoire, and 20.6% (578/2,2804, 119/2,2804 reporting multiple diseases) in Madagascar. Among these HCWs, 22.3% (129/578) in Madagascar, 28.8% (64/222) in Nigeria, 30.0% (21/70) in Côte d'Ivoire and 36.5% (53/145) in Democratic Republic of the Congo reported to take medication, in terms of angiotensin-converting enzyme inhibitors, corticosteroids, non-steroidal anti-inflammatory, or anti-rheumatic drugs.

In Côte d'Ivoire, the odds of at least one chronic disease increased by 4% with every year increase in age (AOR: 1.04; 95% CI: 1.01–1.07) and by 10% with every kg/m^2^ unit increase in BMI (AOR: 1.10; 95% CI: 1.03–1.17) (Table 2A).

**Table 2:** Association between sociodemographic characteristics, risk factors, at least one chronic disease and hypertension in Côte d'Ivoire **(A)**, the Democratic Republic of the Congo **(B)**, in Madagascar **(C)**, and in Nigeria **(D)**.

Covariates		Reported history of any diagnosed chronic disease		Bivariate analysis		Logistic Regression results‘* (*N* = 510)		Reported history of hypertension		Bivariate analysis		Logistic Regression results‘^#^ (*N =* 510)	
		Yes (Row %)	No (Row %)	OR	95% CI	AOR	95% CI	Yes (Row %)	No (Row %)	OR	95% CI	AOR	95% CI
Sex	
	Female	48 (15.9)	254 (84.1)	Ref.				16 (5.3)	286 (94.7)	Ref.			
	Male	22 (10.2)	194 (89.8)	0.60	0.35–1.03			12 (5.6)	204 (94.4)	1.05	0.49–2.27		
Age (median, IQR)		40 (29–47)	34 (28–40)	1.05	1.02–1.08	1.04	1.01–1.07	46 (40–51)	34 (28–41)	1.11	1.06–1.15	1.10	1.05–1.14
Level or Type of Care	
	Primary, non-hospital	40 (12.3)	286 (87.7)	Ref.				17 (5.2)	309 (94.8)	Ref.			
	Secondary	30 (15.6)	162 (84.4)	1.32	0.79–2.20			11 (5.7)	181 (94.3)	1.10	0.51–2.41		
BMI (mean ± SD)		27.1 ± 4.0	25.2 ± 4.2	1.11	1.05–1.18	1.10	1.03–1.17	27.6 ± 3.3	25.3 ± 4.2	1.13	1.04–1.24	1.10	1.00–1.22
Smoking	
	Never smoked	64 (13.5)	409 (86.5)	Ref.				25 (5.3)	448 (94.7)	Ref.			
	Current/past smoker	5 (12.2)	36 (87.8)	0.89	0.34–2.35			2 (4.9)	39 (95.1)	0.92	0.21–4.02		
Profession	
	Administrative staff	15 (17.8)	70 (82.4)	Ref.				7 (8.2)	78 (91.8)	Ref.			
	Medical doctor	5 (16.7)	25 (83.3)	0.93	0.31–2.83			1 (3.3)	29 (96.7)	0.38	0.05–3.26		
	Paramedics higher educ.	27 (15.7)	145 (84.3)	0.87	0.43–1.74			12 (7.0)	160 (93.2)	0.84	0.32–2.21		
	Paramedics lower educ.	13 (8.8)	135 (91.2)	0.45	0.20–1.00			4 (2.7)	144 (97.3)	0.31	0.10–1.09		
	Auxiliary staff	9 (12.3)	64 (87.7)	0.66	0.27–1.60			4 (5.5)	69 (94.5)	0.65	0.18–2.30		
AOR, Adjusted Odds ratio; CI, Confidence interval. ‘Coefficient only displayed for variables kept in final model. ^*^No influential observations were dropped. ^#^No influential observations were dropped.													

Covariates		Reported history of any diagnosed chronic disease		Bivariate analysis		Logistic Regression results‘* (*N =* 1.125)		Reported history of hypertension		Bivariate analysis		Logistic Regression results‘^#^ (*N* = 1.120)	
		Yes (Row %)	No (Row %)	OR	95% CI	AOR	95% CI	Yes (Row %)	No (Row %)	OR	95% CI	AOR	95% CI
Sex	
	Female	89 (14.6)	544 (85.9)	Ref.		Ref.		61 (9.6)	572 (90.4)	Ref.		Ref.	
	Male	56 (9.4)	543 (90.7)	0.63	0.44–0.90	3.04	0.6–15.4	36 (6.0)	563 (94.0)	0.60	0.39–0.92	0.31	0.18–0.55
Age (median, IQR)		50 (42–60)	39 (31–49)	1.06	1.04–1.07	1.09	1.06–1.11	49.5 (42–60)	39 (31–49)	1.06	1.04–1.07	1.31	1.15–1.50
Level or type of care	
	Primary, hospital	38 (9.9)	347 (90.1)	Ref.				20 (5.2)	365 (94.8)	Ref.			
	Primary, non-hospital	81 (12.4)	572 (87.6)	1.29	0.86–1.94			63 (9.7)	590 (90.4)	1.95	1.16–3.28		
	Secondary	26 (13.4)	168 (86.6)	1.41	0.83–2.41			14 (7.2)	180 (92.8)	1.42	0.70–2.88		
BMI (mean ± SD)		24.2 ± 3.4	23.2 ± 3.4	1.09	1.03–1.15	1.07	1.01–1.14	24.5 ± 3.7	23.2 ± 3.4	1.11	1.04–1.18	1.66	1.27–2.18
Interaction Sex # Age						0.96	0.93–0.99						
Interaction Sex # BMI												0.99	0.99–1.00
Smoking	
	Never smoked	130 (12.4)	915 (87.6)	Ref.				88 (8.4)	957 (91.6)	Ref.			
	Current/past smoker	15 (8.4)	163 (91.6)	0.65	0.37–1.13			9 (5.1)	169 (94.9)	0.58	0.29–1.17		
Profession	
	Administrative staff	11 (13.1)	73 (86.9)	Ref.				10 (11.9)	74 (88.1)	Ref.			
	Medical doctor	10 (14.3)	60 (85.7)	1.11	0.44–2.78			6 (8.6)	64 (91.4)	0.69	0.24–2.01		
	Paramedics higher educ.	82 (11.0)	663 (90.0)	0.82	0.42–1.61			54 (7.3)	691 (92.7)	0.58	0.28–1.18		
	Paramedics lower educ.	24 (13.7)	151 (86.3)	1.05	0.49–2.27			16 (9.1)	159 (90.9)	0.74	0.32–1.72		
	Auxiliary staff	18 (11.4)	140 (88.6)	0.85	0.38–1.90			11 (7.0)	147 (93.0)	0.55	0.22–1.36		
AOR, Adjusted odds ratio; CI, Confidence interval; ‘coefficient only displayed for variables kept in final model. ^*^no influential observations were dropped. ^#^Five influential observations were dropped.													

Covariates		Reported history of any diagnosed chronic disease		Bivariate analysis		Logistic Regression results‘* (*N* = 1.910)		Reported history of hypertension		Bivariate analysis		Logistic Regression results‘^#^ (*N* = 2.756)	
		Yes (Row %)	No (Row %)	OR	95% CI	AOR	95% CI	Yes (Row %)	No (Row %)	OR	95% CI	AOR	95% CI
Sex	
	Female	399 (23.2)	1,323 (76.8)	Ref.		Ref.		225 (13.1)	1,497 (86.9)	Ref.		Ref.	
	Male	179 (16.5)	903 (83.5)	0.66	0.54–0.80	0.65	0.51–0.81	92 (8.5)	990 (91.5)	0.62	0.48–0.80	0.53	0.40–0.71
Age (median, IQR)		44 (33–53)	32 (27–42)	1.07	1.06–1.07	1.06	1.05–1.07	50 (41–56)	32 (27–42)	1.11	1.09–1.12	1.11	1.10–1.12
Level or type of care	
	Primary, hospital	28 (14.1)	170 (85.9)	Ref.		Ref.		18 (9.1)	180 (90.9)	Ref.			
	Primary, non-hospital	97 (24.1)	306 (75.9)	1.94	1.22–3.07	1.58	0.96–2.60	60 (14.9)	343 (85.1)	1.76	1.01–307		
	Private	197 (23.2)	653 (76.8)	1.84	1.20–2.83	1.52	0.96–2.40	112 (13.2)	738 (86.8)	1.53	0.90–2.58		
	Secondary	134 (25.3)	396 (74.7)	2.07	1.32–3.22	2.26	1.41–3.63	66 (12.5)	464 (87.6)	1.43	0.83–2.48		
	Tertiary	122 (14.9)	697 (85.1)	1.07	0.69–1.66	1.13	0.70–1.81	61 (7.5)	758 (92.6)	0.81	0.47–1.40		
BMI (mean ± SD)		24.3 ± 3.6	22.8 ± 3.4	1.13	1.10–1.16	1.06	1.03–1.09	25.1 ± 3.4	22.7 ± 3.4	1.19	1.15–1.24	1.13	1.09–1.17
Smoking	
	Never smoked	494 (20.6)	1,905 (79.4)	Ref.				272 (11.3)	2,127 (88.7)	Ref.			
	Current/past smoker	80 (20.1)	317 (79.9)	0.97	0.75–1.27			42 (10.6)	355 (89.4)	0.93	0.66–1.30		
Profession	
	Administrative staff	113 (28.0)	290 (72.0)	Ref.		Ref.		73 (18.1)	330 (81.9)	Ref.			
	Medical doctor	77 (31.6)	167 (68.4)	1.18	0.84–1.67	1.05	0.71–1.54	45 (18.4)	199 (81.6)	1.02	0.68–1.54		
	Paramedics higher educ.	275 (17.9)	1,258 (82.1)	0.56	0.44–0.72	0.87	0.65–1.15	131 (8.6)	1,402 (91.5)	0.42	0.31–0.58		
	Paramedics lower educ.	25 (29.4)	60 (70.6)	1.07	0.64–1.79	1.48	0.83–2.59	13 (15.3)	72 (84.7)	0.82	0.43–1.55		
	Auxiliary staff	87 (16.7)	433 (83.3)	0.52	0.38–0.71	0.67	0.47–0.94	54 (10.4)	466 (89.6)	0.52	0.36–0.77		
AOR, Adjusted Odds ratio, CI, Confidence interval; ‘Coefficient only displayed for variables kept in final model. ^*^No influential observations were dropped. ^#^No influential observations were dropped.													

Covariates		Reported history of any diagnosed chronic disease		Bivariate analysis		Logistic Regression results‘* (*N* = 1,969)		Reported history of hypertension		Bivariate analysis		Logistic Regression results‘^#^ (*N* = 1,904)	
		Yes (Row %)	No (Row %)	OR	95% CI	AOR	95% CI	Yes (Row %)	No (Row %)	OR	95% CI	AOR	95% CI
Sex	
	Female	154 (10.2)	1,351 (89.8)	Ref.				112 (7.4)	1,393 (92.6)	Ref.		Ref.	
	Male	68 (8.7)	716 (91.3)	0.83	0.62–1.12			49 (6.3)	735 (93.8)	0.83	0.59–1.17	0.62	0.40–0.97
Age (median, IQR)		47 (39–53)	37 (31–44)	1.09	1.08–1.11	1.10	1.08–1.12	48 (42–54)	37 (31–44)	1.11	1.09–1.13	1.11	1.08–1.13
Level or type of care	
	Primary non-hospital	33 (8.4)	359 (91.6)	Ref.				22 (5.6)	370 (94.4)	Ref.		Ref.	
	Private	20 (4.0)	480 (96.0)	0.45	0.26–0.80			12 (2.4)	488 (97.6)	0.41	0.20–0.85	0.71	0.32–1.55
	Secondary	71 (13.2)	467 (86.8)	1.65	1.07–2.56			59 (11.0)	476 (89.0)	2.08	1.25–3.46	1.99	1.10–3.60
	Tertiary	94 (11.9)	697 (88.1)	1.47	0.97–2.23			65 (8.2)	726 (91.8)	1.01	0.91–2.48	1.41	0.79–2.53
BMI (mean ± SD)		28.8 ± 5.0	26.8 ± 4.6	1.10	1.06–1.13	1.08	1.04–1.12	29.4 ± 4.8	26.8 ± 4.6	1.12	1.08–1.16	1.07	1.03–1.12
Smoking	
	Never smoked	209 (9.7)	1,952 (90.3)	Ref.				152 (7.0)	2,009 (93.0)	Ref.			
	Current/past smoker	103 (90.4)	11 (9.7)	1.00	0.53–1.89			7 (6.1)	107 (93.9)	0.86	0.40–1.89		
Profession	
	Administrative staff	37 (8.4)	405 (91.6)	Ref.		Ref.		27 (6.1)	415 (93.9)	Ref.		Ref.	
	Medical doctor	30 (17.5)	141 (82.5)	2.33	1.39–3.91	2.16	1.20–3.89	20 (11.7)	151 (88.3)	2.04	1.11–3.74	2.16	1.06–4.41
	Paramedics higher educ.	71 (10.6)	598 (89.4)	1.30	0.86–1.97	1.07	0.67–1.73	50 (7.5)	619 (92.5)	1.24	0.76–2.02	0.98	0.55–1.74
	Paramedics lower educ.	46 (9.4)	442 (90.6)	1.14	0.72–1.79	1.32	0.80–2.19	38 (7.8)	450 (92.2)	1.30	0.78–2.16	1.68	0.92–3.04
	Auxiliary staff	25 (6.3)	373 (93.7)	0.73	0.43–1.24	0.87	0.49–1.57	18 (4.5)	380 (95.5)	0.73	0.39–1.34	1.08	0.55–2.11
AOR, Adjusted odds ratio; CI, Confidence interval; ‘Coefficient only displayed for variables kept in final model. ^*^Five influential observations were dropped. ^#^One influential observations were dropped.													

In the Democratic Republic of the Congo, the odds of at least one chronic disease increased by 9% with every year increase in age (AOR: 1.09; 95% CI: 1.06–1.11) and by 7% with every unit increase of kg/m^2^ in BMI (AOR: 1.07; 95% CI: 1.01–1.14). In females, 1 year change in age had 0.96 times the effect of 1 year change in males (AOR: 0.96; 95% CI: 0.93–0.99) (Table 2B).

In Madagascar, the odds of at least one chronic disease were lower among males (AOR: 0.65; 95% CI: 0.51–0.81), and increased by 6% with every year increase in age (AOR: 1.06; 95% CI: 1.05–1.07) as well as with every kg/m^2^ unit increase in BMI (AOR: 1.06; 95% CI: 1.03–1.09). HCWs working in secondary level of care facilities had increased odds of at least one chronic disease (AOR: 2.26; 95% CI: 1.41–3.63) compared to HCWs working at primary hospitals and auxiliary staff had decreased odds compared to administrative staff (AOR: 0.67; 95% CI: 0.47–0.94).

In Nigeria, the odds of having at least one chronic disease increased by 10% with every year increase in age (AOR: 1.10; 95% CI: 1.08–1.12) and by 8% with every kg/m^2^ unit increase in BMI (AOR: 1.08; 95% CI: 1.04–1.12). Compared to administrative staff, medical doctors had increased odds of at least one chronic disease (AOR: 2.16; 95% CI: 1.20–3.89).

### Prevalence of hypertension and associated risk factors

High blood pressure was the most common self-reported chronic disease across all countries [Côte d'Ivoire 5.4% (28/518), Nigeria 7.0% (161/2.291), Democratic Republic of the Congo 7.9% (97/1.232), and Madagascar 11.3% (317/2804)]; with 12.4% (12/97) (Democratic Republic of the Congo), 25.0% (7/28)(Côte d'Ivoire), 28.0% (45/161) (Nigeria), and 31.2% (99/317)(Madagascar) of affected HCWs in each country reporting to take angiotensin-converting enzyme inhibitors.

In Côte d'Ivoire, the odds of hypertension increased by 10% with every year increase in age (AOR: 1.10; 95% CI: 1.05–1.14) and every kg/m^2^ unit increase in BMI (AOR: 1.10; 95% CI: 1.00–1.22) (Table 2A).

In the Democratic Republic of the Congo, the odds of hypertension were lower among males (AOR: 0.31; 95% CI: 0.18–0.55), and increased by 31% with every year increase in age (AOR: 1.31; 95% CI: 1.15–1.50) as well as by 66% with every kg/m^2^ unit increase in BMI (AOR: 1.66; 95% CI: 1.27–2.18) (Table 2B).

In Madagascar, the odds of hypertension were lower among males (AOR: 0.53; 95% CI: 0.40–0.71), increased by 11% with every year increase in age (AOR: 1.11; 95% CI: 1.10–1.12) and by 13% with every kg/m^2^ unit increase in BMI (AOR: 1.13; 95% CI: 1.09–1.17) (Table 2C).

In Nigeria, the odds of hypertension were lower among males (AOR: 0.62; 95% CI: 0.40–0.97), increased by 11% with every year increase in age (AOR: 1.11; 95% CI: 1.08–1.13) and by 7% with every kg/m^2^ unit increase in BMI (AOR: 1.07; 95% CI: 1.03–1.12) (Table 2D). Medical doctors showed increased odds of hypertension (AOR: 2.16; 95% CI: 1.06–4.41) compared to administrative staff. Overall as well, HCWs working at secondary level compared to HCWs working at primary level of care similarly had increased odds of hypertension (AOR: 1.99; 95% CI: 1.00–3.60).

## Discussion

In this multisite cross-sectional survey in Côte d'Ivoire, Democratic Republic of the Congo, Madagascar, and Nigeria, we found alarming prevalence estimates of potentially untreated chronic diseases among HCWs, with hypertension being the most prevalent condition. The prevalence of self-reported hypertension and at least one chronic disease varied across these countries, with HCWs in Madagascar reporting the highest figures for both outcomes. Overall, older age and higher BMI increased the odds of self-reported hypertension and chronic diseases across all countries. For public health planning, knowledge about the risk profile and more specifically knowledge about individual and occupational risk factors is crucial.

Our study adds evidence to the broader discourse on the dual burden of communicable and non-communicable diseases in low-income countries. In addition to studies from Ghana, Mozambique, and Zimbabwe, we found a considerable prevalence of NCDs in four other sub-Saharan Africa countries underlining the current epidemiological transition and the need to encompass NCDs in these settings (15–17).

The self-reported prevalence of hypertension among HCWs in the present study is lower than previously reported for HCWs in West Africa (19). Under-diagnosis of chronic conditions is a well-known and highly prevalent phenomenon. A nationally representative study in the United States, found that less than one third of people with hypertension are aware of their condition (33). Considering this low level of awareness and at the same time the high level of health care in the United States, we can only imagine that this iceberg phenomenon is even more pronounced in the African setting. Applying this phenomenon to our context, self-reported rates potentially only reflect one third of the real prevalence. Under-diagnosis ultimately leads to under-treatment. In the current study, less than one third of HCWs suffering from hypertension took angiotensin-converting enzyme inhibitors. This potential undertreatment could lead to important implications for public health planning, as NCDs are the leading cause of deaths with devasting consequences in general, and hypertension causing stroke, heart attack, and renal failure (34).

In our study, age was associated with any chronic disease and hypertension across all study sites. Age is a well-known risk factor for the development of any NCD, not only because the prevalence of behavioral risk factors, such as insufficient physical activity and hyperlipidemia increases with age (35) but also because of biological risk factors, such as dyslipidemia (36). Population growth and aging can even partly explain the increase in the proportion of total disability-adjusted life years (DALYs) attributable to NCDs within the “Global Burden of Disease Study” (14).

In addition to age as a risk factor for NCDs, in our study, female gender was also found to be a risk factor. The relation between gender and the development of NCDs is still not clearly understood. While there are studies that confirm male gender as a risk factor in sub-Saharan Africa (19, 37–40), women often face limited access to healthcare and at the same time experience higher exposure to other NCD risk factors due to gender norms and intersecting determinants such as education (41). In addition, there is a known gender gap in self-reported health, as women across the globe more frequently report poorer health (42). This potential mix between biological factors, as well as societal factors underlines the need for gender-specific research, preventive measures, and management for HCWs in the study locations.

Apart from non-modifiable risk factors such as age and sex, BMI was found to be significantly associated with self-reported hypertension and chronic disease across all study sites. BMI is a known risk factor also from other settings, to which death and DALYs from NCDs can be largely attributed (43, 44). HCWs in Africa are prone to the increasing “obesogenic” environment that is attributable to less physical activity and increasing consumption of calorie-dense foods, especially in urban areas (19). Cultural practices and perception of beauty in some African areas also could promote obesity among female HCWs. For example, “fattening room” is commonly practiced in Nigeria, which increase obesity and consequently the risk of diabetes (45).

In terms of occupational risk factors, the current study found medical doctors and HCWs working at secondary level to have higher odds of reporting NCDs. Contrarily, auxiliary workers showed lower odds of suffering from any NCDs. These odds might be influenced by the socioeconomic position. A recent systematic review by the World Health Organization reported that socioeconomic positions affects behavioral risk factors in low-income and lower-middle-income countries. These behavioral risk factors in higher socioeconomic groups can translate in less physically activity and increased consumption of fat, salt and processed food (46). A study from Ghana confirms tertiary education and private employment as risk factors for NCDs (15). However, evidence was not conclusive, as lower socioeconomic status was also associated with higher prevalence of tobacco and alcohol use (46).

In addition, medical doctors are an extremely scarce workforce in the study settings, leading to high levels of stress. Not only during the COVID-19 pandemic, when high levels of burnout were reported (47). These high levels of stress can in turn also influence the risk of NCD development (48). In settings where health system capacity and HCWs are already scarce, HCW absenteeism from work due to chronic diseases could have a large impact on health system resilience. Given that the wellbeing of HCWs has direct impact on the health of the general population, this shortage of HCWs can potentially delay the global attainment of the SDGs. Targeted interventions for early screening of NCDs and reduction of burden among HCWs in the study countries are therefore urgently needed. However, achieving this will depend on improving HCWs’ access to NCD screening tools and diagnostics in addition to appropriate treatment, with special focus on the vulnerable groups such as older women. The way forward could involve health education of HCWs, health checkups at the workplace, as well as administrative decisions to improve the safety of hospital environments (49).

Providing evidence to help protect HCWs, our study emphasizes the need for a comprehensive socio-cultural approach for developing and implementing interventions to address gaps in knowledge and health seeking behaviors as well as the promotion of NCD screening in the context of occupational and health promotion services for HCW. This approach can inform policy instruments and national programs for managing occupational health and safety of health workers, that currently 169 WHO member states lack.

### Strengths and limitation

Our study contributed to the scant literature on NCDs among HCWs in sub-Saharan Africa, with generalizability of the findings particularly enhanced by the large sample size from the four included countries. However, the study has some limitations. Our data were collected in a larger study to assess the seroprevalence of SARS-CoV-2 and vaccine effectiveness. Therefore, the design of the study was not tailored to assess NCDs, and tools such as the WHO STEPwise approach or other standardized NCD surveillance tools were not utilized (50). We collected self-reported sociodemographic data, basic medical history information, and risk behaviors, including tobacco use, but not data on factors such as diet or physical activity, socioeconomic status and anthropometric indicators (e.g., waist circumference) or laboratory indicators (e.g., plasma glucose). Unlike the objective screening of hypertension and other chronic diseases on a regular basis, our self-reported outcome of NCDs and factors such as weight and height to determine BMI are subject to recall and social desirability bias. The data reported on treatment is limited to medications that were discussed to influence the susceptibility or severity of COVID-19 and is therefore not including all first-line treatments for hypertension nor all other NCDs. Our questionnaire was not specifically tested for validity or reliability. In order to limit the impact on its reliability, we tested the questionnaire for comprehension, piloted in a sub-sample and trained healthcare personnel across all study sites. The initially planned stratified multi-stage random sampling had to be adapted due to logistical and security concerns in each of the countries, leading to different strategies across countries, which we did not account for in the analysis. Numbers for HCWs working in every HCF were not available across all study sites, therefore, the strobe diagram cannot be fully described, but sampling fractions based on district level can be provided, whereby numbers for private healthcare facilities had to be projected in Madagascar and Nigeria. The logistic model on any chronic disease in the Democratic Republic of the Congo shows some level of misspecification in the linktest. However, applying the probit regression, as an equivalent for practical purposes (51), this is no longer the case. For consistency, odds ratios of the logit model are displayed for the Democratic Republic of the Congo. Lastly, our analysis loses some strength due to the fact that given the heterogeneity of data, no model was run including multi-site data.

### Conclusion

The prevalence of at least one self-reported chronic disease or hypertension among HCWs varied across Côte d'Ivoire, the Democratic Republic of the Congo, Madagascar, and Nigeria with less than 37% HCWs reported being on treatment for both conditions. Hypertension, when not controlled can have devasting consequences such as stroke, heart attack, and renal failure (34). These findings call for increased awareness and underline the importance of addressing these needs among HCWs—one of the main building blocks of the health systems. Moreover, the uncontrolled burden of hypertension and NCDs in general will pose major challenges to the health system. Sub-Saharan African countries need to prioritize the diagnosis of chronic disease, including hypertension and the management of these conditions through appropriate treatment. We identified several individual (age, sex, and BMI), as well as occupational (profession and level of healthcare) factors that significantly influenced the odds of NCDs. These factors should be considered in a comprehensive socio-cultural approach for developing and implementing interventions targeting NCDs among HCWs, particularly in national health plans to counteract the current and rising burden of untreated NCDs across sub-Saharan Africa.

## Data availability statement

Data can be shared based on reasonable request to the corresponding author, and after agreement with all country authorities.

## Ethics statement

The studies involving humans were approved by ethics committee of Berlin Charité (Number EA2/248/21) and from national ethics committees of each partner country (CIV: 216-21/MSHPCMU/CNESVS-km, DRC: ESP/CE/22B/2022, MDG: 12 MSANP/SG/AMM/CERBM, and NG: NHREC/01/01/207-29/11/2021).The studies were conducted in accordance with the local legislation and institutional requirements. The participants provided their written informed consent to participate in this study.

## Author contributions

SM: Data curation, Formal analysis, Investigation, Methodology, Validation, Writing – original draft, Writing – review & editing, Conceptualization, Funding acquisition, Project administration, Resources. KE: Data curation, Formal analysis, Methodology, Writing – original draft, Writing – review & editing, Investigation, Project administration. JR: Data curation, Investigation, Methodology, Writing – original draft, Writing – review & editing, Formal analysis, Project administration. FR: Data curation, Investigation, Methodology, Writing – original draft, Writing – review & editing, Formal analysis, Project administration. FM: Data curation, Investigation, Methodology, Writing – original draft, Writing – review & editing, Formal analysis, Project administration. LB: Methodology, Writing – original draft, Writing – review & editing, Data curation, Investigation, Formal analysis, Project administration. SoT: Data curation, Methodology, Writing – original draft, Writing – review & editing, Formal analysis, Investigation, Project administration. IB: Data curation, Formal analysis, Writing – original draft, Writing – review & editing, Investigation, Methodology, Project administration. LR: Data curation, Methodology, Writing – original draft, Writing – review & editing, Formal analysis, Investigation, Project administration. SN: Data curation, Methodology, Writing – original draft, Writing – review & editing, Formal analysis, Investigation, Project administration. GO: Data curation, Methodology, Writing – original draft, Writing – review & editing, Formal analysis, Investigation, Project administration. FP: Data curation, Methodology, Writing – original draft, Writing – review & editing, Formal analysis, Investigation, Project administration. SaT: Data curation, Methodology, Writing – original draft, Writing – review & editing, Formal analysis, Investigation, Project administration. TE: Data curation, Methodology, Writing – original draft, Writing – review & editing, Formal analysis, Investigation, Project administration. TO: Data curation, Methodology, Writing – original draft, Writing – review & editing, Formal analysis, Investigation, Project administration. CA-K: Writing – original draft, Writing – review & editing, Data curation, Investigation, Project administration. BD: Writing – original draft, Writing – review & editing, Data curation, Investigation, Project administration. RZ: Writing – original draft, Writing – review & editing, Data curation, Investigation, Project administration. SA: Writing – original draft, Writing – review & editing, Data curation, Investigation, Project administration. CO: Writing – original draft, Writing – review & editing, Data curation, Investigation, Project administration. CE-B: Data curation, Formal analysis, Funding acquisition, Methodology, Resources, Supervision, Visualization, Writing – original draft, Writing – review & editing, Conceptualization, Investigation, Project administration, Validation.

## References

[1] WHO (2022). Fact sheets noncommunicable disease. Available at: https://www.who.int/news-room/fact-sheets/detail/noncommunicable-diseases (Accessed October 31, 2022).

[2] WHO (2024). Premature mortality from noncommunicable disease. Available at: https://www.who.int/data/gho/indicator-metadata-registry/imr-details/3411 (Accessed February 23, 2024).

[3] Bigna JJ Noubiap JJ. The rising burden of non-communicable diseases in sub-Saharan Africa. Lancet Glob Health. (2019) 7:e1295–6. doi: 10.1016/S2214-109X(19)30370-5 31537347

[4] WHO (2022). The Global Health Observatory. Available at: https://www.who.int/data/gho/data/themes/topics/indicator-groups/indicator-group-details/GHO/sdg-target-3.4-noncommunicable-diseases-and-mental-health (Accessed October 31, 2022).

[5] Sovova E Nakladalová M Kaletova M Sovova M Radova L Kribska M. Which health professionals are most at risk for cardiovascular disease? Or do not be a manager [journal article]. Int J Occup Med Environ Health. (2014) 27:71–7. doi: 10.2478/s13382-014-0228-1 24488775

[6] Dutheil F Baker JS Mermillod M De Cesare M Vidal A Moustafa F. Shift work, and particularly permanent night shifts, promote dyslipidaemia: a systematic review and meta-analysis. Atherosclerosis. (2020) 313:156–69. doi: 10.1016/j.atherosclerosis.2020.08.015, PMID: 33069952

[7] Owoeye O Tomori A Akinbo S. Pedometer-determined physical activity profile of healthcare professionals in a Nigerian tertiary hospital [original article]. Niger Med J. (2016) 57:99–103. doi: 10.4103/0300-1652.182070, PMID: 27226683 PMC4872499

[8] Nilan K McKeever TM McNeill A Raw M Murray RL. Prevalence of tobacco use in healthcare workers: a systematic review and meta-analysis. PLoS One. (2019) 14:e0220168. doi: 10.1371/journal.pone.0220168, PMID: 31344083 PMC6657871

[9] WHO (2022). Occupational health: health workers. Available at: https://www.who.int/news-room/fact-sheets/detail/occupational-health--health-workers (Accessed Feb 23, 2022).

[10] Pryor L Da Silva MA Melchior M. Mental health and global strategies to reduce NCDs and premature mortality. Lancet Public Health. (2017) 2:e350–1. doi: 10.1016/S2468-2667(17)30140-8, PMID: 29253472

[11] Chen J Farah N Dong RK Chen RZ Xu W Yin J. Mental health during the COVID-19 crisis in Africa: a systematic review and meta-analysis. Int J Environ Res Public Health. (2021) 18. doi: 10.3390/ijerph182010604, PMID: 34682357 PMC8536091

[12] WHO (2017). What needs to be done to solve the shortage of health workers in the African Region. Available at: https://www.afro.who.int/news/what-needs-be-done-solve-shortage-health-workers-african-region (Accessed November 1, 2017).

[13] WHO (2016). Health workforce requirements for universal health coverage and the sustainable development goals. Human Resources for Health Observer, 17.

[14] Gouda HN Charlson F Sorsdahl K Ahmadzada S Ferrari AJ Erskine H. Burden of non-communicable diseases in sub-Saharan Africa, 1990–2017: results from the global burden of disease study 2017. Lancet Glob Health. (2019) 7:e1375–87. doi: 10.1016/S2214-109X(19)30374-2, PMID: 31537368

[15] Konkor I Kuuire VZ. Epidemiologic transition and the double burden of disease in Ghana: what do we know at the neighborhood level? PLoS One. (2023) 18:e0281639. doi: 10.1371/journal.pone.0281639, PMID: 36827236 PMC9956066

[16] Nyabani P. Epidemiological transition and the dual burden of communicable and noncommunicable diseases in Zimbabwe. Int J Noncommun Dis. (2021) 6:166. doi: 10.4103/jncd.jncd_69_21

[17] Ciccacci F Orlando S Majid N Marazzi C. Epidemiological transition and double burden of diseases in low-income countries: the case of Mozambique. Pan Afr Med J. (2020) 37:49. doi: 10.11604/pamj.2020.37.49.23310, PMID: 33209176 PMC7648489

[18] Mudie K Jin MM Tan KL Addo J Dos-Santos-Silva I Quint J. Non-communicable diseases in sub-Saharan Africa: a scoping review of large cohort studies. J Glob Health. (2019) 9:020409. doi: 10.7189/jogh.09.020409, PMID: 31448113 PMC6684871

[19] Bosu WK. The prevalence, awareness, and control of hypertension among workers in West Africa: a systematic review. Glob Health Action. (2015) 8:26227. doi: 10.3402/gha.v8.26227, PMID: 25623611 PMC4306751

[20] Osei-Yeboah J Kye-Amoah KK Owiredu WKBA Lokpo SY Esson J Bella Johnson B. Cardiometabolic risk factors among healthcare workers: a cross-sectional study at the Sefwi-Wiawso municipal hospital, Ghana. Biomed Res Int. (2018) 2018:8904548. doi: 10.1155/2018/8904548, PMID: 29850585 PMC5937588

[21] Onyemelukwe O Kaoje Y Isa B Mamza A Iyanda M Bello-Ovosi B. Pre-obesity/obesity in relation to blood pressure among tertiary healthcare workers in an African setting. African J Endocrinol Metab. (2022) 12:28–40. doi: 10.4103/ajem.ajem_12_22

[22] Skaal L Pengpid S. Obesity and health problems among south African healthcare workers: do healthcare workers take care of themselves? S Afr Fam Pract. (2011) 53:563–7. doi: 10.1080/20786204.2011.10874153

[23] JICA (2022). Data Collection Survey on Health Sector Policy for Universal Health Coverage toward Women, Children and Lower Income People in Ivory Coast Final Report.

[24] Observataory SM (2022). Madagascar's health system 2022. Available at: https://www.severemalaria.org/madagascars-health-system (Accessed 12 July 2023).

[25] Federal Government of Nigeria (2018–2022). Second National Strategic Health Development Plan [NHSDP II]. 2017. 1–136 p.

[26] WHO (2019). Classifying health workers: mapping occupations to the international standard classification. Available at: https://www.who.int/activities/improving-health-workforce-data-and-evidence (Accessed March 20, 2019).

[27] Boynton PM Greenhalgh T. Selecting, designing, and developing your questionnaire. BMJ. (2004) 328:1312–5. doi: 10.1136/bmj.328.7451.1312, PMID: 15166072 PMC420179

[28] Bukhman G Mocumbi AO Atun R Becker AE Bhutta Z Binagwaho A. The lancet NCDI poverty commission: bridging a gap in universal health coverage for the poorest billion. Lancet. (2020) 396:991–1044. doi: 10.1016/s0140-6736(20)31907-3, PMID: 32941823 PMC7489932

[29] Hartung C Lerer A Anokwa Y Tseng C Brunette W Borriello G (2010). “Open data kit: tools to build information services for developing regions” in *Proceedings of the 4th ACM/IEEE International Conference on Information and Communication Technologies and Development*. London, United Kingdom. Association for Computing Machinery. Article 18.

[30] Wickham H FR Henry L Müller K Vaughan D (2023). A grammar of data manipulation. Available at: https://dplyr.tidyverse.org; https://github.com/tidyverse/dplyr

[31] R Core Team R (2013). R: A language and environment for statistical computing.

[32] Paul P Pennell ML Lemeshow S. Standardizing the power of the Hosmer–Lemeshow goodness of fit test in large data sets. Stat Med. (2013) 32:67–80. doi: 10.1002/sim.5525 22833304

[33] Wall HK Hannan JA Wright JS. Patients with undiagnosed hypertension: hiding in plain sight. JAMA. (2014) 19:1973–4. doi: 10.1001/jama.2014.15388 PMC4596255 25399269

[34] Messerli FH Williams B Ritz E. Essential hypertension. Lancet. (2007) 370:591–603. doi: 10.1016/s0140-6736(07)61299-9 17707755

[35] Sapkota BP Baral KP Rehfuess EA Parhofer KG Berger U. Effects of age on non-communicable disease risk factors among Nepalese adults. PLoS One. (2023) 18:e0281028. doi: 10.1371/journal.pone.0281028, PMID: 37267282 PMC10237426

[36] Cho SMJ Lee HJ Shim JS Song BM Kim HC. Associations between age and dyslipidemia are differed by education level: the cardiovascular and metabolic diseases etiology research center (CMERC) cohort. Lipids Health Dis. (2020) 19:12. doi: 10.1186/s12944-020-1189-y, PMID: 31954396 PMC6969451

[37] Bosu WK. Determinants of mean blood pressure and hypertension among Workers in West Africa. Int J Hypertens. (2016) 2016:3192149. doi: 10.1155/2016/3192149, PMID: 26949543 PMC4754493

[38] Russell JB Rahman-Sesay J Conteh V Conteh S Jalloh AP Ibrahim-Sayo E. Prevalence, awareness and risk factors of hypertension among health Workers at the Connaught Teaching Hospital, Sierra Leone. West Afr J Med. (2020) 37:450–9. PMID: 33058119

[39] Twagirumukiza M De Bacquer D Kips JG de Backer G Stichele RV Van Bortel LM. Current and projected prevalence of arterial hypertension in sub-Saharan Africa by sex, age and habitat: an estimate from population studies. J Hypertens. (2011) 29:1243–52. doi: 10.1097/HJH.0b013e328346995d, PMID: 21540748

[40] van de Vijver S Akinyi H Oti S Olajide A Agyemang C Aboderin I. Status report on hypertension in Africa--consultative review for the 6th session of the African union conference of ministers of health on NCD's. Pan Afr Med J. (2013) 16:38. doi: 10.11604/pamj.2013.16.38.3100, PMID: 24570798 PMC3932118

[41] NCD Alliance Women and NCDs. (2023). Available at: https://ncdalliance.org/why-ncds/ncds-and-sustainable-development/women-and-ncds

[42] Boerma T Hosseinpoor AR Verdes E Chatterji S. A global assessment of the gender gap in self-reported health with survey data from 59 countries. BMC Public Health. (2016) 16:675. doi: 10.1186/s12889-016-3352-y, PMID: 27475755 PMC4967305

[43] Fuentes R Nilson E Rezende LFM Christofaro DGD Silva DR Ferrero-Hernández P. Future burden of non-communicable diseases attributable to overweight in Chile: a multistate life table modeling study. BMC Public Health. (2023) 23:1337. doi: 10.1186/s12889-023-16255-w, PMID: 37438831 PMC10337135

[44] Felisbino-Mendes MS Cousin E Malta DC Machado ÍE Ribeiro ALP Duncan BB. The burden of non-communicable diseases attributable to high BMI in Brazil, 1990–2017: findings from the global burden of disease study. Popul Health Metrics. (2020) 18:18. doi: 10.1186/s12963-020-00219-y, PMID: 32993699 PMC7525961

[45] Enang O. The fattening rooms of Calabar—a breeding ground for diabesity. Diabet Voice. (2009)

[46] Allen L Williams J Townsend N Mikkelsen B Roberts N Foster C. Socioeconomic status and non-communicable disease behavioural risk factors in low-income and lower-middle-income countries: a systematic review. Lancet Glob Health. (2017) 5:e277–89. doi: 10.1016/s2214-109x(17)30058-x, PMID: 28193397 PMC5673683

[47] Fathuse N Hlongwana KW Ginindza TG. "why am I even Here if I Can't save the patients?": the frontline healthcare Workers' experience of burnout during COVID-19 pandemic in Mthatha, South Africa. Int J Environ Res Public Health. (2023) 20:5451. doi: 10.3390/ijerph20085451, PMID: 37107733 PMC10138325

[48] Fricchione GL. The challenge of stress-related non-communicable diseases. Med Sci Monit Basic Res. (2018) 24:93–5. doi: 10.12659/msmbr.911473, PMID: 29904042 PMC6035498

[49] Joseph B Arasu S. Occupational risks and health promotion for healthcare workers In: Kickbusch I Ganten D Moeti M, editors. Handbook of Global Health. Cham: Springer International Publishing (2021). 2581–608.

[50] WHO (2023). WHO STEPwise approach to surveillance. Available at: https://www.who.int/europe/tools-and-toolkits/who-stepwise-approach-to-surveillance (Accessed April 27, 2023).

[51] Gelman A Hill J. Data Analysis Using Regression and Multilevel/Hierarchical Models. New York: Cambridge University Press (2006).

